# Extensive structural variations between mitochondrial genomes of CMS and normal peppers (*Capsicum annuum* L.) revealed by complete nucleotide sequencing

**DOI:** 10.1186/1471-2164-15-561

**Published:** 2014-07-04

**Authors:** Yeong Deuk Jo, Yoomi Choi, Dong-Hwan Kim, Byung-Dong Kim, Byoung-Cheorl Kang

**Affiliations:** Department of Plant Science, Plant Genomics and Breeding Institute, and Institute of Green BioScience and Technology, Seoul National University, Seoul, 151-921 South Korea; Advanced Radiation Technology Institute, Korea Atomic Energy Research Institute, Jeongeup, 580-185 South Korea; Department of Molecular Biosciences, The University of Texas at Austin, Austin, TX 78712 USA

## Abstract

**Background:**

Cytoplasmic male sterility (CMS) is an inability to produce functional pollen that is caused by mutation of the mitochondrial genome. Comparative analyses of mitochondrial genomes of lines with and without CMS in several species have revealed structural differences between genomes, including extensive rearrangements caused by recombination. However, the mitochondrial genome structure and the DNA rearrangements that may be related to CMS have not been characterized in *Capsicum* spp.

**Results:**

We obtained the complete mitochondrial genome sequences of the pepper CMS line FS4401 (507,452 bp) and the fertile line Jeju (511,530 bp). Comparative analysis between mitochondrial genomes of peppers and tobacco that are included in Solanaceae revealed extensive DNA rearrangements and poor conservation in non-coding DNA. In comparison between pepper lines, FS4401 and Jeju mitochondrial DNAs contained the same complement of protein coding genes except for one additional copy of an *atp6* gene (ψ*atp6-2*) in FS4401. In terms of genome structure, we found eighteen syntenic blocks in the two mitochondrial genomes, which have been rearranged in each genome. By contrast, sequences between syntenic blocks, which were specific to each line, accounted for 30,380 and 17,847 bp in FS4401 and Jeju, respectively. The previously-reported CMS candidate genes, *orf507* and ψ*atp6-2,* were located on the edges of the largest sequence segments that were specific to FS4401. In this region, large number of small sequence segments which were absent or found on different locations in Jeju mitochondrial genome were combined together. The incorporation of repeats and overlapping of connected sequence segments by a few nucleotides implied that extensive rearrangements by homologous recombination might be involved in evolution of this region. Further analysis using mtDNA pairs from other plant species revealed common features of DNA regions around CMS-associated genes.

**Conclusions:**

Although large portion of sequence context was shared by mitochondrial genomes of CMS and male-fertile pepper lines, extensive genome rearrangements were detected. CMS candidate genes located on the edges of highly-rearranged CMS-specific DNA regions and near to repeat sequences. These characteristics were detected among CMS-associated genes in other species, implying a common mechanism might be involved in the evolution of CMS-associated genes.

**Electronic supplementary material:**

The online version of this article (doi:10.1186/1471-2164-15-561) contains supplementary material, which is available to authorized users.

## Background

Mitochondrial genomes of higher plants are clearly different from their animal counterparts and from plastid genomes in terms of evolutionary dynamics of genome structure [1, 2]. Although the rate of synonymous substitution in plant mitochondrial DNA (mtDNA) is 50–100 times and three times lower than in vertebrate mtDNA and plant plastid DNA, respectively, structural variations including changes in gene order, rearrangement, genome expansion and shrinkage, and incorporation of foreign DNAs are more common in plant mitochondria compared to the others [3–5]. The complexity of the plant mtDNA structure has been attributed to existence of a reservoir of low-copy-number subgenomic mtDNA molecules suppressed via nuclear control as well as the presence of repeat sequences dispersed throughout the genome that can mediate recombination [6–8].

Structural variations in mtDNA are associated with several mutant phenotypes such as cytoplasmic male sterility (CMS) and variegated-leaf phenotypes [9–11]. CMS has been studied in many crop species because of its economic importance for hybrid seed production. Most identified CMS-associated genes are novel chimeric open reading frames (ORFs) generated by fusion of several sequence segments due to rearrangement of the mitochondrial genome [9, 12]. Although CMS-associated genes in different crop species do not show significant similarity in their sequences, most share several features such as possession of a transmembrane domain and co-transcription with normal mitochondrial genes, which often encode ATP-synthase or cytochrome C oxidase subunits [9, 13–15]. The detailed mechanisms of how these genes originated remain unknown, however.

Complete sequencing and comparative analysis of mtDNA of normal and CMS lines has been performed in several crop species including sugar beet (Owen-type CMS), maize (CMS-T, CMS-S, CMS-C), wheat (K-type CMS), rice (CW- and LD-type CMS), rapeseed (pol- and nap-type CMS), and radish (Ogura- and DCGMS-type CMS), revealing structural variation in mtDNA and identifying genes responsible for CMS [16–24]. These studies showed that mitochondrial genome structures in lines exhibiting CMS are extensively rearranged compared to those of fertile lines, whereas gene contents are mostly conserved [12]. For example, in sugar beet, normal and CMS lines have mitochondrial genomes composed of different arrays of fourteen sequence blocks that are syntenic between the two genomes [23]. Recently, Tanaka et al. [24] showed that the mitochondrial genome of a radish with CMS, Ogura cytoplasm, has a large CMS-specific DNA region in addition to syntenic block sequences. This region, containing the CMS-associated *orf138*, was postulated to be inserted into the fertile mitochondrial genome by recombination via inverted repeat sequences located on its borders. However, the origin of the CMS-associated gene and other CMS-specific sequences in this region are still unknown.

CMS has been widely used in hybrid seed production in chili peppers. Only a single source of cytoplasm has been reported to be responsible for CMS [25]. Kim et al. [14, 26] found two candidate CMS-associated genes, *orf456* and ψ*atp6-2*. The *orf456* gene fused with a mitochondrial target sequence induced male sterility in transgenic Arabidopsis [14]. In later studies, it was shown that the *orf456* gene exists as a longer *orf* named *orf507* [27]. Further analysis of the function of ORF507 protein showed that the interaction of ORF507 with an ATP-synthase subunit (ATP synthase 6 kDa subunit) may cause impaired ATP synthase activity in CMS cytoplasm [28]. The ψ*atp6-2* gene is the truncated form of *atp6-2* that was generated by rearrangement in the 3′ region of *atp6-2*. Differences in the transcription pattern of ψ*atp6-2* between male-sterile and restorer lines demonstrated a possible association of this gene with CMS [26]. However, complete sequence analyses of mitochondrial genomes to elucidate CMS-specific mtDNA structures and their evolutionary history has not been performed in *Capsicum*.

In this study, we first report the complete mitochondrial genome sequences for *Capsicum*. Comparative analysis between CMS and normal mitochondrial genomes provides insights into the evolution of mitochondrial genome structure associated with CMS in plants.

## Methods

### Plant materials

A pepper CMS line, ‘FS4401’ (S/*rfrf*), and a restorer line, ‘Jeju’ (N/*RfRf*), which are known to contain CMS and normal cytoplasm, respectively, were provided by Monsanto Korea. ‘FS4401’ is a breeding line containing a natural CMS cytoplasm that has been used as the stable CMS source in seed companies in Korea and ‘Jeju’ is a landrace in Korea. For each pepper line, approximately 3,000 seedlings were grown in dark conditions for twenty days and harvested to isolate mitochondria.

### Mitochondrial DNA extraction

The method described by Millar et al. [29] and modified by Kim [14] was used for mitochondrial DNA extraction. Seedlings were homogenized using a mortar with isolation buffer consisting of 0.3 M mannitol, 50 mM Tris–HCl, 3 mM EDTA, 1 mM 2-mercaptoethanol, 0.1% BSA, 1% PVP, and protease inhibitor cocktail (Roche Applied Science, Indianapolis, USA) and adjusted to pH 7.5 with KOH. Homogenized tissue was filtered with one layer of Miracloth and four layers of cheesecloth. Two rounds of centrifugation at 2,000 g for 10 min were then performed to remove cell debris and larger organelles in cells. The supernatant subsequently was centrifuged at 15,000 g for 10 min to obtain a crude mitochondrial pellet. The pellet was gently resuspended with a painter’s brush in isolation buffer without PVP, adjusted to 10 mM MgCl_2_ and treated with DNase I (50 μg/ml) for one hour to degrade nuclear DNA. The sample was adjusted to 20 mM EDTA and centrifuged at 15,000 g for 10 min. The pellet was gently resuspended in 500 μL buffer II (0.3 M sucrose, 0.05 M Tris–HCl, 0.02 M EDTA, 0.1% BSA, pH 7.5) with a painter’s brush. After resuspension, the sample was layered on a 30-mL Percoll cushion (28% Percoll, 0.3 M sucrose, 0.05 M Tris–HCl, 0.02 M EDTA, pH 7.5) and centrifuged at 40,000 g for 90 min. The yellowish mitochondrial ring in the middle of the cushion was collected. The mitochondrial fraction was rinsed with washing buffer (0.3 M mannitol, 50 mM Tris–HCl, 1 mM EDTA, pH7.5) using three rounds of centrifugation at 15,000 g for 10 min. Mitochondrial DNA was extracted following the method described by Kim [30].

### DNA sequencing

mtDNA sequencing was performed using the GS-FLX system (Roche Applied Science, Indianapolis, USA) and the resultant single-read sequences were assembled by Newbler Assembler Software Version 2.0 (454 Life Sciences, Branford, USA) in the National Instrumentation Center for Environmental Management (NICEM, Seoul, Republic of Korea) (Additional file 1).

### Sequence assembly

Contigs were further assembled using the following strategies. First, analysis using the Basic Local Alignment Search Tool (BLAST) was performed against the Genbank nucleotide database (http://www.ncbi.nlm.nih.gov) to obtain mitochondrial contig sequences longer than 1 kb. The contig sequences that contained significant matches to known mtDNA sequences from other species were used for the next analyses. In the second step, a DNA library in which the average size of inserts was about 3 kb in length was constructed and the end sequences of inserts were analyzed using the ABI3700 sequencing system (Applied Biosystems, Foster City, USA) in NICEM (Seoul, Republic of Korea; Additional file 1). The mate-pair information of insert end sequences obtained by ABI sequencing was used to order contig sequences. In the third step, primers were designed from end sequences of each contig and all possible combinations of primers were tested by PCR analysis to identify connected contigs. If a gap sequence obtained from PCRs contained only plastid-derived sequence, the gap was considered to be obtained due to contamination with plastids during mitochondrial DNA preparation, not because of the existence of subgenomic mtDNA molecules. Through this step, gaps could be closed and the number of contigs was reduced. In the fourth step, genome walking from the ends of each assembled scaffold sequence was conducted using the GenomeWalker™ universal kit (Clontech, Mountain View, USA) according to the manufacturer’s instructions. In the fifth step, LA-PCRs were performed to fill remaining gaps between scaffolds using TaKaRa LA Taq™ (TaKaRa, Shiga, Japan). Finally, all information obtained by the stepwise approach was used to construct a master circle model that contained at least one copy of every mtDNA contig. The contig sequences that could be connected to multiple other contig sequences were regarded as repeated sequences. These sequences could be contained in a master circle two or more than two times. Insertions of repeated sequence were validated by PCR with primers designed from flanking regions of repeated sequences.

### Accession numbers of mitochondrial genome sequences

Complete mitochondrial genome sequences of FS4401 (CMS) and Jeju (male-fertile) have been deposited in the GenBank nucleotide sequence database under the accession numbers of KJ865409 and J865410, respectively.

### Screening a CM334 bacterial artificial chromosome library

BAC clones containing *cox2* or *atp6* was screened from a 12× BAC library of CM334, which is a Mexican landrace of chili pepper (*C.annuum* L.) containing normal cytoplasm [31]. The *cox2* and *atp6* genes of CM334 were amplified from total DNA of CM334 using primer sets designed from sugar beet mitochondrial DNA (GenBank accession number: BA000009). The amplicons were labeled and used as the probes for BAC library screening based on hybridization as described by Yoo et al. [31]. The sequences of the selected BAC clones containing both *cox2* or *atp6* was analyzed by 12× Shot-gun sequencing, which was carried out in NICEM (Seoul, Republic of Korea).

### Gene annotation and characterization of ORFs

The protein and rRNA genes on mtDNA sequences were identified using BLAST with the nucleotide and protein database in GenBank (http://www.ncbi.nlm.nih.gov). The tRNA genes were identified using the tRNA scan-SE program (http://lowelab.ucsc.edu/tRNAscan-SE/). ORFs that were predicted to encode hypothetical proteins longer than 100 amino acids were screened using custom-made Perl scripts. The presence of a transmembrane domain in each hypothetical protein was predicted using TMHMM server v.2.0 (http://www.cbs.dtu.dk/services/TMHMM/).

### Sequence comparison and repeat sequence analysis

Alignment between target sequences was performed using the BLASTN algorithm (http://blast.ncbi.nlm.nih.gov/). Pools of repeated sequences were obtained by analysis using BLASTN in which a given target sequence was used as both query and subject sequence. The alignments that met the criteria to be syntenic sequence blocks or repeated sequences in terms of length and similarity were isolated and visualized as Scalable Vector Graphics using custom-made Perl scripts followed by manual modification.

## Results

### Assembly of complete mitochondrial genome sequences

We obtained contigs containing most mitochondrial genome information for a pepper CMS line (FS4401) and a fertile line (Jeju) by sequencing their mitochondrial genomes using the 454 GS-FLX system. However, it appeared that too many mtDNA contigs (>1 kb) were obtained for each line when the high coverage of sequencing (>100×) was considered (Additional file 1). The reasons for this were revealed in the process of further assembly of contigs and analysis of gap sequences. Firstly, contamination with plastid DNA hampered sequence assembly at the positions of mitochondrial genomes where plastid-derived sequences were located. Secondly, ends of large repeated sequences remained unconnected due to the short length of individual reads in the 454 GS-FLX system (Additional file 1). Therefore, we constructed a DNA library containing inserts averaging 3 kb in length and analyzed the end sequences of inserts using ABI3700 for ordering of contigs (Additional file 1). In addition, we performed PCR analysis and genome walking from contig ends to test all of the possible combinations of large repeated sequences with other contig sequences. The final circular molecules for complete mitochondrial genomes included every mtDNA contig longer than 1 kb at least one time and were consistent with all of the results produced during sequence assembly process.

Meanwhile, screening of a bacterial artificial chromosome (BAC) library of pepper line ‘CM334’ with normal (fertile) cytoplasm using *cox2* and *atp6* as probes resulted in the isolation of one BAC clone containing both *cox2* and *atp6*. The complete sequence of this BAC clone (74,615 bp) was obtained by shotgun sequencing and assembly.

### Comparative analysis of general features and sequence contents between mitochondrial genomes

General features of mitochondrial genomes were compared between FS4401, Jeju, and tobacco (Table 1). Tobacco is the only species in the Solanaceae for which complete mtDNA sequence has been reported [32]. The complete mitochondrial genomes of FS4401 and Jeju were 507,452 and 511,530 bp in length, respectively, significantly larger than that of tobacco (Table 1). The proportion of protein-coding sequences was similar between pepper mtDNAs, at 7.9% and 7.7%, respectively, while it was higher in tobacco due to repetition of some genes (*trnM, rrn26, nad2a, sdh3*) and smaller genome size. The ratio of chloroplast-derived sequences was slightly higher in Jeju (12.7%) than FS4401 (11.8%), and those values were about 2.5 times higher than in tobacco (4.5%). Tobacco had a larger proportion of repeated sequences, mainly due to containing larger repeat sequences. The total length of repeat sequences was longer in Jeju than in FS4401. The contents of genes encoding proteins and rRNAs were the same between Jeju and tobacco, whereas FS4401 had an additional copy of the *atp6* gene, named ψ*atp6-2* [26]. The number of genes encoding tRNAs were different between mtDNAs. FS4401 had one and four additional tRNA genes of chloroplast origin compared to Jeju and tobacco, respectively.

**Table 1:** **General features of mitochondrial genomes of two pepper lines and one tobacco line**

Features	FS4401	Jeju	***Nicotiana tabacum***
Genome size (bp)	507,452	511,530	430,597
GC content (%)	44.5	44.6	45.0
Coding sequences (bp)^a^	40,085 (7.9%)	39,524 (7.7%)	43,642 (10.1%)
Plastid-derived sequences (bp)^b^	59,873 (11.8%)	64,815 (12.7%)	19,492 (4.5%)
Repeated sequences (bp)^c^	42,505 (8.4%)	70,122 (13.7%)	73,511 (17.1%)
Gene content (number)	66	64	61
Protein coding genes^d^	38	37	37
rRNAs	3	3	3
tRNAs^e^	25 (13)	24 (12)	21 (9)

^a^All of the copies of duplicated genes were included.

^b^Complete sequences of chloroplast genomes of FS4401 [33] and *Nicotiana tabacum* [34] were used to screen for plastid-derived sequences in mitochondrial genomes of FS4401/Jeju and *N. tabacum*, respectively, based on the BLASTN algorithm. The value for *N. tabacum* was different from that of Sugiyama et al. [35] probably due to differences in methodology used to isolate plastid-derived sequence.

^c^Sequences longer than 100 bp and showing similarity higher than 95% between copies were considered repeated. In case repeated sequence units were overlapping, nucleotides that were included in repeated sequences at least one time were counted without repetition.

^d^ *rpl10*, which was reported to be a conserved mitochondrial gene by Kubo and Arimura [32] was added to the list of genes described by Sugiyama et al. in tobacco [35].

^e^The number of tRNA genes included in plastid-derived sequences is given in parenthesis.

The tobacco line is ‘Bright Yellow 4′ for which the mitochondrial genome was analyzed by Sugiyama et al. [35].

Sequence alignment analysis showed that most sequence content was shared between two mtDNAs of pepper. 95.1% of the sequence of FS4401 could be aligned with Jeju and 98.0% of Jeju with FS4401. In comparative analysis with tobacco sequence, only about 46.3% and 45.4% of each genome could be aligned with tobacco mtDNA, respectively (Additional file 2).

### Distribution of sequence blocks syntenic between pepper and tobacco mitochondrial genomes

**Figure 1:**
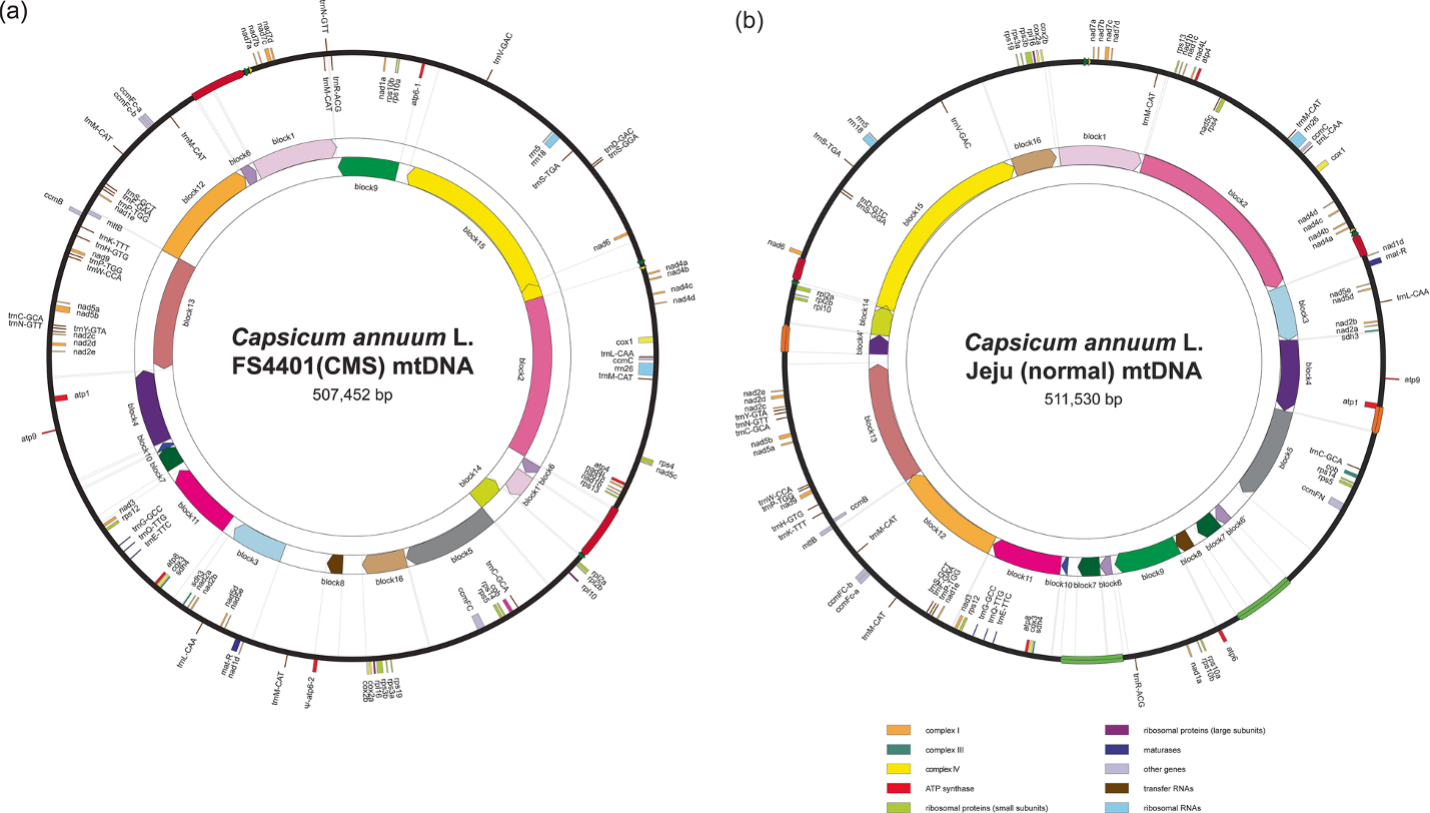
**Gene maps of the mitochondrial genomes of CMS and male-fertile pepper lines. (a)** Gene map of FS4401 (CMS) **(b)** Gene map of Jeju (male-fertile). The genes drawn outside of the circle are transcribed clockwise and those inside, counterclockwise. The colors of the genes denote the functions of the gene products. Large repeat sequences (>1 kb) are shown as colored arrows on the outer circle. Sequence blocks that were syntenic between genomes (>2 kb; > 95% similarity) are depicted on the inner circles. They were drawn in two lines of inner circles to separate blocks in different directions.

Sequence blocks between mitochondrial genomes of the two pepper lines and the tobacco line were defined based on similarity higher than 95% and matching length longer than 2 kb. These sequence blocks were then localized on each genome (Figure 1; Additional file 3). In the alignment between FS4401 and tobacco, a total of 33 sequence blocks that covered 116,598 bp of the FS4401 mitochondrial genome were detected. The comparison between Jeju and tobacco accounted for 122,124 bp of the Jeju mitochondrial genome and revealed the same 33 blocks as well as two additional sequence blocks including one located downstream of *cox2* and another on which *nad9*, *trnP*, *trnW*, and *ccmB* were localized. Most of the syntenic sequence blocks contained clusters of genes, resulting in high conservation of the gene clustering pattern between pepper and tobacco. However, four of the tobacco gene clusters, including *atp9-rps13-nad1bc*, *nad4-rps1-nad5ab, nad3-nad1a,* and *rps4-nad6*, were not conserved in FS4401 and Jeju. The order of the syntenic blocks was extremely different among the mitochondrial genomes, demonstrating extensive rearrangement between non-coding regions.

### Gene contents and localization on mitochondrial genomes

We next annotated and localized the genes with known functions on the FS4401 and Jeju mitochondrial genomes (Figure 1). The protein-coding genes were classified according to the functions of proteins; the 37 protein-coding genes shared by FS4401 and Jeju included nine genes for complex I proteins (*nad1, nad2, nad3, nad4, nad4L, nad5, nad6, nad7, nad9*), two for complex II (*sdh3, sdh4*), one for complex III (*cob*), three for complex IV (*cox1, cox2, cox3*), five for ATP synthase subunits (*atp1, atp4, atp6, atp8, atp9*), ten for ribosomal proteins (*rpl2, rpl5, rpl10, rpl16, rps3, rps4, rps10, rps12, rps13, rps19*), four for proteins involved in cytochrome c biogenesis (*ccmB, ccmC, ccmFc, ccmFN*), one for maturase (*matR*), and one for a protein translocation system subunit (*mttB*). In addition to these genes, FS4401 had another copy of the *atp6* gene (ψ*atp6-2*). Both FS4401 and Jeju contained three rRNA genes (*rrn5, rrn18, rrn26*), while FS4401 contained one additional tRNA gene compared to Jeju.

**Figure 2:**
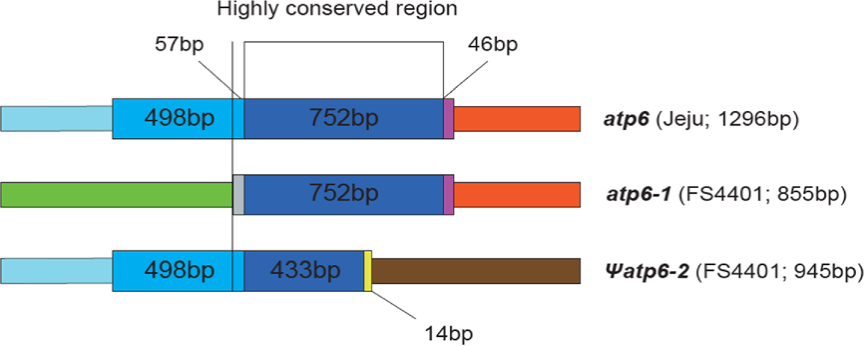
**Structure of the** ***atp6*** **gene copies in Jeju and FS4401.** The sequences correspond to gene-coding region are drawn as the wider rectangles and the upstream or downstream regions as the narrower bars. The sequence units that show high similarity (>99%) to each other and included in the same category of sequence characteristics (non-coding region/coding region, *atp6* region showing high conservation/poor conservation among plant taxa) are depicted as the same color. The overall scheme of figure was adopted from Kim et al. [26].

Comparison of protein-coding gene sequences between FS4401 and Jeju revealed six genes exhibiting polymorphism in their nucleotide sequences (Table 2). Sequence polymorphisms in *atp4, atp8, rpl2, sdh3*, and *atp6* resulted in a change in protein sequence, whereas a synonymous substitution was detected in *matR*. Changes in the gene product length were predicted for *atp4, atp8,* and *rpl2* due to in-frame indels, whereas length polymorphism in *atp6* was attributable to a structural rearrangement (Figure 2). The *atp6* gene in Jeju showed high similarity with *atp6-1* of FS4401 over three-fifths of the gene, spanning 800 bp including the highly conserved region of *atp6* genes [26], whereas the sequence upstream of the conserved region could be aligned with the additional copy of *atp6* in FS4401, ψ*atp6-2*. The nucleotide sequence and the structure of the gene-flanking regions of *atp6* of Jeju were different from any *atp6* reported in previous research where two copies of *atp6* genes were present even in normal cytoplasm [26]. Comparison of the genes carrying nucleotide sequence polymorphism between the pepper species to the corresponding genes in tobacco showed that the polymorphic sites of *matR* and *sdh3* in FS4401 were the same as those of tobacco, indicating that nucleotide substitutions in these genes were probably not related to sequence alteration during the evolution of CMS. By contrast, differences in the sequences of *atp4*, *atp8*, *rpl2*, and *atp6* of FS4401 relative to those of both Jeju and tobacco pointed to the possibility that variations in these genes might underlie CMS (Table 2).

**Table 2:** **Differences between Jeju and FS4401 in sequences of known genes**

Genes in Jeju	Gene length in Jeju	Polymorphism in FS4401	Corresponding sequence in tobacco
*matR*	1977	904 gcA (A) → 904 gcG (A)	904 gcG (A)
*atp4*	597	16 acGAATATGCAg (T*NMQ*)	16 acGAATATGCAg (TNMQ)
		→ 16 acg (T)	
*atp8*	462	178 ccCAACAGTTTg (P*NSL*)	178 ccCAACTGTTTg (PN*C*L)
		→ 178 ccg (P)	
*rpl2*	999	337 ccCGGGAAGGGggat (P*GKG*D)	337 ccCGGGAAGGGggat (PGKGD)
		→ 337 ccggat (PD)	
*sdh3* ^a^	333	178 tTCttc (*F*F)	178 tCTttc (*S*F)
		→ 178 tCTttc (*S*F)	
*atp6*	1296	ψ*atp6-2*	Higher similarity with *atp6-1* in FS4401
		283 gGt (G) → gCt (A)	
		316 ACa (T) → CAa (Q)	
		454 aaAGaa (KE) → aaCCaa (NQ)	
		no similarity in downstream of 931^th^ bp due to DNA rearrangement	
		*atp6-1*	
		no similarity in upstream of 497^th^ bp due to DNA rearrangement	

^a^The SNP polymorphism can potentially be eliminated if the plant mitochondrial.

C-to-U RNA editing occur.

The pattern of base changes and corresponding amino acid changes is described. The polymorphic site (the number indicates the position of the first nucleotide), with the polymorphic nucleotide capitalized, and the corresponding amino acid in parentheses, is given to the left of the arrow for Jeju and to the right of the arrow for FS4401.

A large number of genes were located close to each other, forming gene clusters that might be co-transcriptional units. In total, sixteen clusters were detected in FS4401 and Jeju, including *rps10ab-nad1a, rrn18-rrn5, trnD-trnS, trnM-rrn26-ccmC-trnL, rps4-nad5c, rps13-nad1bc-nad4L-atp4, rpl2ab-rpl10, rpl5-rps14-cob-trnC, rps19-rps3ab-rpl16-cox2ab, nad1d-matR, sdh3-nad2ab, atp8-cox3-sdh4, nad3-rps12, trnC-trnN-trnY-nad2cde, nad9-trnP-trnW, trnS-trnF-trnP-nad1e* (Figure 1, Additional file 3). Although numerous rearrangements between the two pepper mitochondrial genomes were detected, the clustering pattern of these genes was highly conserved.

### Rearrangements of genome structure between CMS and normal mtDNA

**Figure 3:**
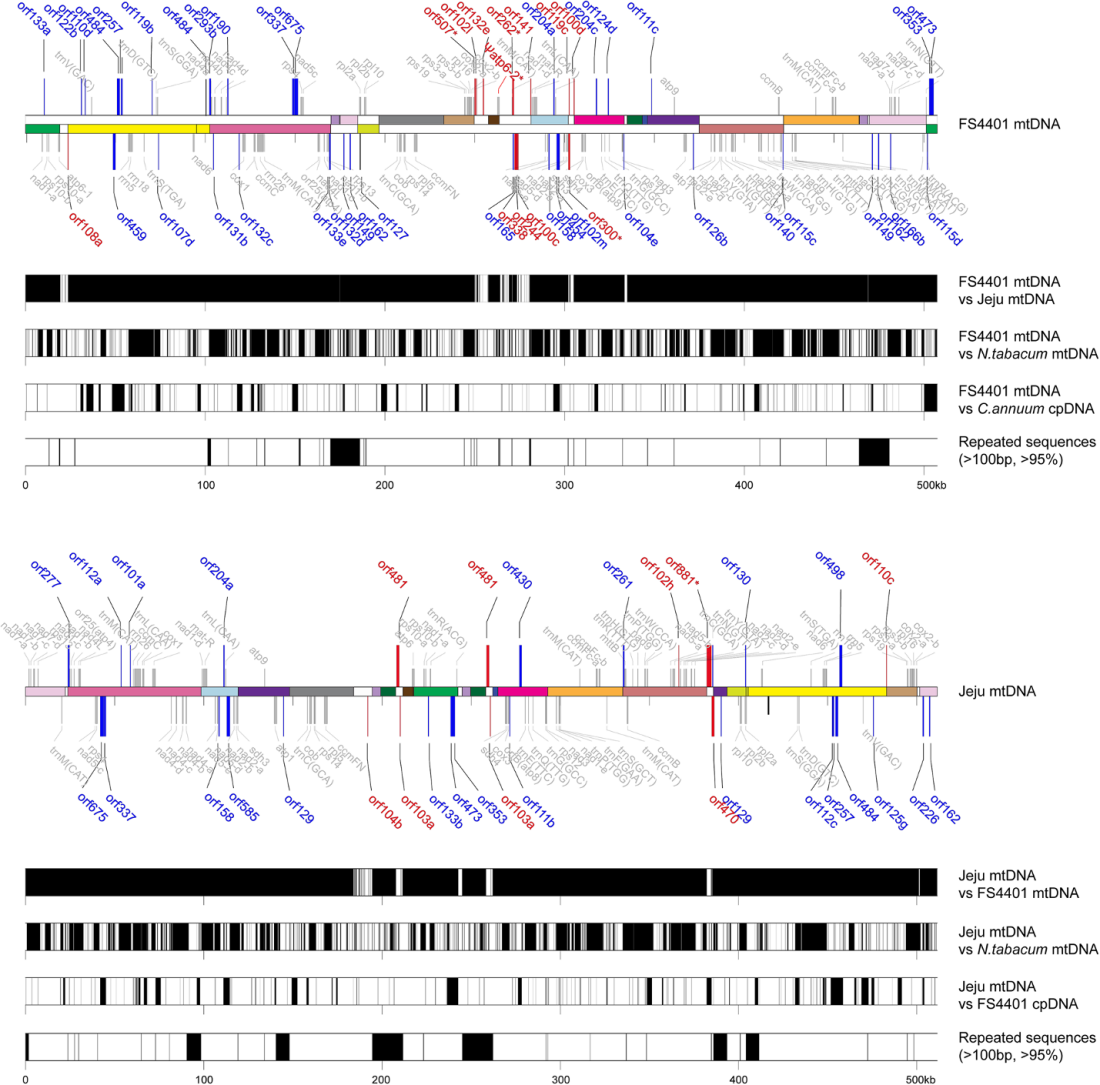
**Distribution of specific** ***ORF*** **s, sequences showing similarity with the other pepper mtDNA, tobacco mtDNA and FS4401 plastid genome, repeated sequences in FS4401 and Jeju mtDNA.** Locations of *ORF*s (longer than 300 bp) that are specific to FS4401 (above) or Jeju (below) are shown on FS4401 or Jeju mtDNA, respectively. Red-colored *ORF*s are specifically present only in one of genomes or carry structural rearrangements. Blue-colored *ORF*s show polymorphism in length or sequence compared to its counterpart. Known genes are depicted in grey. The sequences showing similarity between genomes were determined based on alignment generated using default parameters of the BLASTN algorithm and is depicted by black rectangles or bars. The distribution of repeated sequences in each genome (>100 bp; > 95%) is depicted with black bars and rectangles. The name of *ORF*s indicates the number of amino acids in encoded proteins except for the case of ‘*orf507*’ for which the number of nucleotides in the *ORF* was adopted to name the *ORF* in consistent with the previous research [27].

A total of sixteen syntenic blocks were localized on each genome (Figure 1, Additional files 4 and 5). The sizes of blocks ranged from 2.9 kb (block 10) to 78.9 kb (block 15; Additional files 4 and 5). Block 6 and a part of block 1, block 7 and parts of blocks 4 and 6 were duplicated in FS4401 and Jeju, respectively (Figure 1, Additional files 4 and 5). The mitochondrial genome of FS4401 had a total of eighteen junctions between blocks, whereas Jeju had nineteen. In FS4401, sequences overlap between blocks were detected in seven junctions while no matching sequences between blocks were found at eleven junctions (Additional file 4). Among sequences located between blocks, the sequences between blocks 16 and 8, and between blocks 8 and 3 were noticeable, accounting for 70.5% of the unique sequence of FS4401 mtDNA. The *orf507* and ψ*atp6-2* genes known to be responsible for CMS [14, 26] were localized at the 5′ junctions of the sequence segments between blocks 16 and 8, and blocks 8 and 3, respectively (Figure 3). In Jeju, a total of nine sequences overlapped by adjacent blocks and ten sequences between blocks were detected (Additional file 5). The sequence between blocks 5 and 6′ contained the largest portion (37.7%) of sequences unique to Jeju. Most of the large sequence segments between blocks remained specific to each mtDNA in alignment analysis with less strict criteria (BLASTN default) and also could not be aligned with tobacco mtDNA sequence (Figure 3). A sequence segment between blocks 13 and block 4′ in Jeju contained a chloroplast-derived sequence specific to Jeju (Figure 3). Such sequence overlaps between syntenic blocks in one mitochondrial genome corresponded to repeated sequences located at the edges of blocks in the other genome. The sizes of this kind of repeated sequence varied from 6 to 7,413 bp (Additional files 4 and 5).

End points of several sequence blocks syntenic between FS4401 and Jeju were located very close (<2 kb) to the edges of blocks shared by each pepper line and tobacco analyzed using the same criteria (>2 kb, > 95%; Additional file 3). There were eight and seven block end regions included in this category in FS4401 and Jeju, respectively. At least two rearrangement events, including one during speciation between tobacco and pepper and another during evolution of pepper mitochondrial genomes, were expected in these regions. No single sequence blocks that could cover adjacent ends of blocks syntenic between FS4401 and Jeju were detected in the alignment with tobacco mtDNA except for two syntenic sequence block that were specific to the alignment between Jeju and tobacco. This syntenic block connected the 3′ junction of block 16, which was downstream of *cox2*, and the 5′ junction of block 1 (Additional file 3).

### *ORF*s unique to each mitochondrial genome

We next identified novel open reading frames predicted to encode proteins longer than 100 amino acids in length in the mitochondrial genomes. A total of 155 and 142 *ORF*s (excluding *ORF*s for known genes) were detected in FS4401 and Jeju, respectively. Comparative analysis of these *ORF*s showed that 45 and 30 *ORF*s had polymorphisms with *ORF* counterparts or were specific to one genome in FS4401 and Jeju, respectively (Additional file 6). FS4401 mtDNA contained 33 *ORF*s with SNPs or length polymorphism and 12 *ORF*s that were chimeric, whereas Jeju mtDNA contained 22 polymorphic and 8 chimeric *ORF*s. When the localization of *ORF*s that were chimeric or unique to FS4401 was investigated to search for candidate CMS-associated genes, seven *ORF*s including *orf100d, orf102l, orf108a, orf119c, orf141, orf300* and *orf507* were found to be close (<2 kb) to the edge of sequence blocks syntenic between FS4401 and Jeju (>2 kb; > 95% similarity). When we searched for *ORF*s located near (<2 kb) repeat sequences (>100 bp; > 95% similarity between copies) and containing putative transmembrane domains, six (*orf102l, orf119c, orf262, orf300, orf338, orf507*) and three (*orf262, orf300, orf507*) *ORF*s were identified, respectively (Figure 3). The *orf507* gene, a strong candidate CMS-associated gene reported in previous research [14], met the conditions for candidate genes for CMS. Although the other previously reported candidate, ψ*atp6-2*, was not classified as a novel *ORF* because it was defined as a gene, it also satisfied all of the conditions described above (proximity to edges of syntenic sequence blocks and repeated sequence, containing putative transmembrane domains). Other than these two, only *orf300* showed the same characteristics.

### Structure of sequences around *orf507*and ψ*atp6-2*

**Figure 4:**
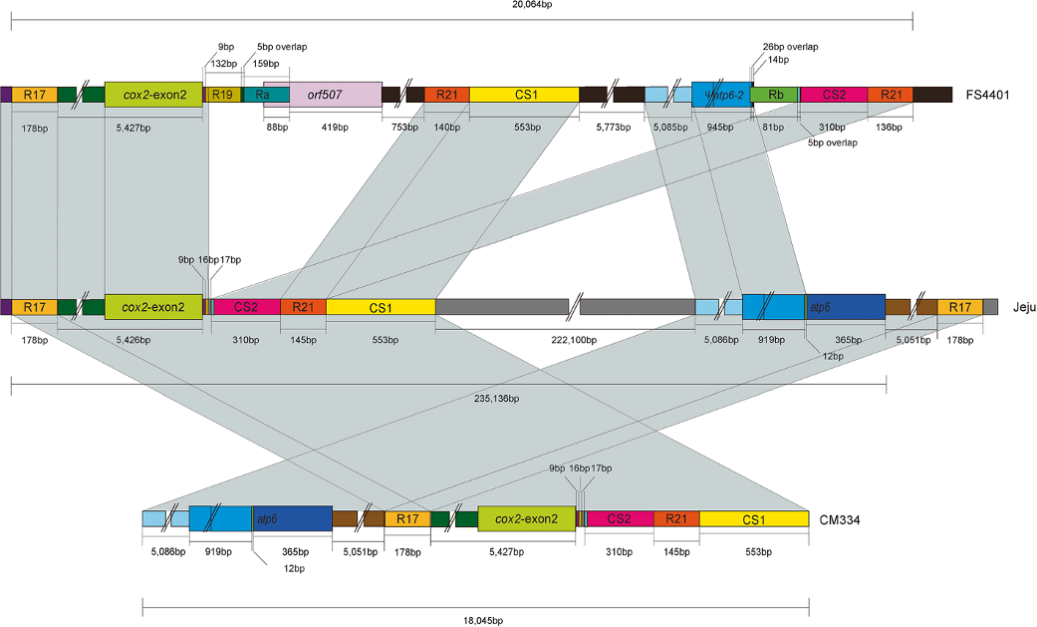
**Comparison of sequence structure around** ***orf507*** **and** ψ***atp6-2*** **between FS4401, Jeju, and CM334.** The sequence blocks conserved between two lines are depicted in the same colors.

The genomic region around *orf507* and ψ*atp6-2* was found to be highly specific to the mitochondrial genome of the line showing CMS in this study as well as previous researches [14, 26]. Therefore, the structure of this DNA region was analyzed in detail (Figure 4). The *orf507* gene was located downstream of *cox2*, and ψ*atp6-2* was about 12 kb from *orf507* in FS4401. In Jeju, however, not only was *orf507* absent, but also *cox2* and *atp6* were distantly located from each other, implying that rearrangements occurred between the two genes. In FS4401, two repeated sequences (R19, Ra) and *orf507*, which were overlapped subsequently by small number of nucleotides, were located downstream of the *cox2.* Sequences showing high similarity to Ra were detected in FS4401, Jeju and tobacco at the 5′ upstream region of the *nad9* gene. The part of *orf507* not covered by Ra showed no similarity to other sequences in FS4401, Jeju, or tobacco mtDNA, nor to any sequences registered in GenBank (http://www.ncbi.nlm.nih.gov/genbank). However, in Jeju, downstream region of *cox2* consisted of sequence elements, CS2, R21, and CS1 that were located on different regions in FS4401*.* DNA region around *atp6-2* (or ψ*atp6-2*) also showed extensive DNA rearrangements. Upstream and 5′ regions of ψ*atp6-2* showed high similarity with the corresponding region in Jeju. However, a repeated sequence Rb and CS2-R21 sequence element composed FS4401-specific DNA structure on downstream of ψ*atp6-2.* The conserved part of ψ*atp6-2,* Rb, and CS2 sequence elements were overlapped subsequently by small number of nucleotides. Stretches of sequences present only in FS4401 were also detected on the region between *orf507* and ψ*atp6-2* and the downstream of ψ*atp6-2.*

The short sequence elements that were detected on and ψ*atp6-2* in FS4401 were present as repeat sequence only in FS4401, implying that these sequences were duplicated during rearrangement (Additional file 7). In particular, R21 in Jeju was duplicated downstream of ψ*atp6-2* in FS4401 resulting in generation of a repeat pair around the ψ*atp6-2* gene. The *orf507* gene and other related sequence elements seemed to be inserted between *cox2* and *R21* via multiple DNA rearrangements.

Comparison of mtDNA around *cox2* and *atp6* in Jeju with corresponding regions in CM334, which is a *C.annuum* landrace introduced from distant area (Mexico) and contains normal cytoplasm, showed that the DNA rearrangement involving R17 resulted in close proximity of *cox2* and *atp6* to each other although sequence contents flanking the two genes were highly conserved. In addition, the gene order of *cox2* and *atp6* was opposite in CM334 compared to FS4401 (Figure 4).

### DNA rearrangement pattern and localization of CMS-associated genes in mitochondrial genomes of other crop species

**Figure 5:**
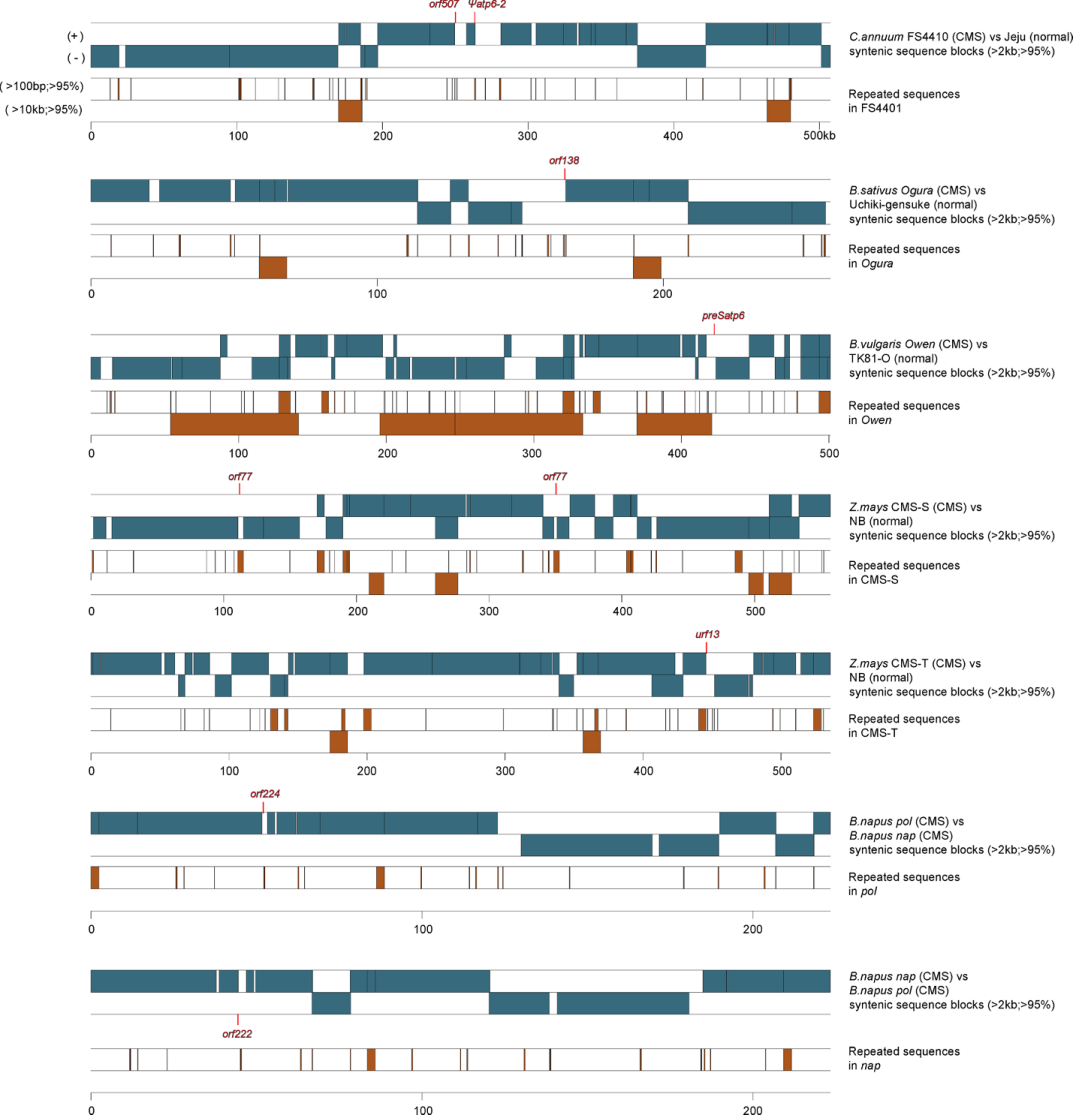
**Localization of syntenic sequence blocks of mitochondrial genomes in other crops.** Sequence blocks showing synteny (>2 kb, > 95%) between a CMS line and a different line were depicted as blue-green color. mtDNAs of CMS lines were used as the reference genome in each comparison. Distribution of repeated sequences (>100 bp, > 95%) in CMS lines is shown with brown bars and boxes. The CMS-associated genes in each crop are indicated above the alignments. Sequence blocks and repeated sequences are depicted in two layers to show the direction and length.

The rearrangement pattern of mitochondrial genomes was investigated in pepper and other crop species including *Brassica sativus*, *Beta vulgaris*, *Zea mays*, and *Brassica napus* for which complete sequences of a CMS-associated mitochondrial genome and at least one mitochondrial genome from different cytoplasm were available and for which the genes responsible for CMS were identified (Figure 5). Alignment of CMS-associated mtDNA with available mtDNA from a different source for seven mitochondrial genomes identified numerous syntenic sequence blocks (>2 kb; > 95%), as reported by many other studies [16, 18, 21–24]. All of the genes known to be associated with CMS were localized close to the edge of syntenic sequence blocks. In particular, CMS-associated genes in pepper and radish were near the end of long sequences located between syntenic blocks. Analysis of repeat sequences (>100 bp; > 95%) showed that CMS genes were always located near or in the repeat sequences.

## Discussion

Here, we report the complete mitochondrial genome sequence of pepper (*C. annuum* L.). This represents only the second mitochondrial genome from the Solanaceae to be fully sequenced, following that of tobacco [35]. Therefore, the mtDNA sequence of pepper described herein is a valuable resource for studying the evolution of mitochondrial genomes in the Solanaceae. The contents and sequences of protein-coding genes were mostly conserved between tobacco and pepper mtDNA although small number of SNPs and indels were found in two and three genes, respectively. It was noticeable that the frequency of in-frame indel polymorphisms was higher in pepper than other crops such as rice [17] and radish [24]. However, the overall structure and the non-coding sequences were extensively changed, resulting in less than 50% coverage of pepper mitochondrial genome sequence by tobacco mtDNA. Similar widespread rearrangements within a plant family have been reported from comparative analysis of Arabidopsis and rapeseed mitochondrial genomes [19], in which only one-third of Arabidopsis and two-third of rapeseed mtDNA could be aligned to each other. By contrast, small DNA regions containing clusters of gene sequences were mostly protected from rearrangement events. The conservation of gene sequence clusters might reflect a requirement for co-regulation of gene expression on each cluster. However, rearrangements were detected even in the small number of gene clusters, including *atp9-rps13-nad1bc*, *nad4-rps1-nad5ab, nad3-nad1a,* and *rps4-nad6*, that have been reported to be putative co-transcribed units in tobacco [35]. In particular, co-transcription of gene cluster *nad3-nad1a* has been confirmed experimentally in tobacco [36, 37], whereas we found that *nad3* and *nad1a* were incorporated into different clusters in pepper mtDNA. Therefore, a change in co-transcription units has resulted from DNA rearrangement during speciation or independent evolution of tobacco and pepper mitochondrial genomes after speciation.

Numerous rearrangements of mitochondrial DNA were also detected even in the comparison of CMS-associated and normal mtDNA within *C. annuum* species. Conservation of gene coding sequences and clustering patterns indicated that maintenance of clusters may be essential for normal expression of genes or those sequences in transcribed regions have characteristics that efficiently suppress rearrangement. However, multiple rearrangements occurring outside of gene clusters resulted in the fragmentation of alignment units between genomes. Several sequence blocks that were syntenic between genomes contained overlapping repeat sequences that might mediate homologous recombination and result in genome rearrangement. However, many sequence blocks were connected with sequences unique to each genome or had overlapping sequences that were shorter than 50 bp which was known as the lower limit of homologous sequence length required for recombination that mediates double-strand break repair [7, 38, 39]. This might be explained by lose of larger repeats during evolution after rearrangements occurred or involvement of nonhomologous end-joining (NHEJ) and/or microhomology-mediated recombination. Comparative analyses on mitochondrial genomes of Arabidopsis ecotypes and mutants revealed that DNA rearrangement results from nonhomologous end-joining (NHEJ) and asymmetric recombination via intermediate-sized repeats followed by randomly occurring double-strand breaks of DNA [7, 40]. Sequences lacking homology were joined by NHEJ, while asymmetric recombination is accompanied by repeat sequences longer than 50 bp [7]. Recombinations via microhomologous repeats (ranging from 6 to 31 bp) have been also reported in pearl millet and maize mutants showing nonchromosomal stripe (NCS) phenotype [41–43]. Microhomology-mediated break-induced replication (MMBIR) have been proposed as one of the mechanism for microhomology-mediated rearrangements in plastids and mitochondria [44, 45].

A significant number of ends of sequence blocks syntenic between FS4401 and Jeju were located close to the junctions of sequence blocks syntenic between FS4401 and tobacco or Jeju and tobacco. Therefore, those regions experienced at least two rearrangement events within a very short distance: one between pepper and tobacco, and the other between CMS and normal pepper lines. Why recombination frequently occurs in specific regions is still unknown although localization of DNA cruciforms, localized melting due to high transcriptional activity, and stalling replication folks have been suggested as possible explanations [40, 46]. Further investigation on a large number of mitochondrial genome sequences in diverse plant families is required to identify and characterize recombination hotspots.

The *orf507* and ψ*atp6-2* genes are known to be associated with CMS in pepper, based on genetic and functional analyses [14, 26, 28]. Comparison between complete mtDNA sequences from CMS-associated and normal cytoplasm in this study reinforced these genes as CMS candidate genes. Although a large number of *ORF*s were specific to the CMS-associated mitochondrial genome, only one *ORF* (*orf300*; Figure 3) in addition to *orf507* and ψ*atp6-2* had the typical characteristics shared by most CMS-associated genes in other species: formation of the *ORF* by novel DNA rearrangement [9], presence of a transmembrane domain [12], and localization close to a junction of syntenic sequence blocks and repeat sequences (discussed below). However, the potential association of other screened *orf*s including *orf300* with CMS also needs to be investigated because discrepancies in cytoplasm types and haplotypes of markers based on *orf507* and ψ*atp6-2* have been reported in several germplasms [47, 48], which may due to incorrect identification of candidate CMS genes or to the existence of a different CMS source.

The genomic regions around *orf507* and ψ*atp6-2* were clearly distinguished from other DNA regions because they were included among the largest sequence fragments highly specific to the CMS line and matched no other known sequences. Recently, similar results were reported for radish *Ogura* type cytoplasm in which the CMS-associated gene *orf138* was located on an edge of the largest genomic region unique to the CMS line [24]. Although the insertion of the DNA region containing *orf138* could be demonstrated to result from homologous recombination using a pair of inverted repeat sequences on the ends, the region around *orf507* and ψ*atp6-2* in pepper contained more complicated structure, hampering the elucidation of the mechanism by which the genomic structure arose. The origin of the 3′ part of *orf 507* and a large portion of the region around *orf507* and ψ*atp6-2* remains unknown. One possible explanation for how these sequences came to be specifically present in the CMS line might be substoichiometric shift (SSS), which has been reported in several plant species [6, 41, 49, 50]. According to the SSS model, subgenomic molecules of mtDNA are present at very low copy number under normal conditions, in which recombination of intermediate-sized repeat sequences is suppressed, and if this recombination is activated (e.g., under certain conditions), these molecules can be efficiently amplified by recombination-dependent replication and maintained as the predominant form of subgenomic molecules even in subsequent generations [40]. In fact, small amounts of *orf507* and ψ*atp6-2* were detected by PCR even in fertile pepper lines [47]. Therefore, the subgenomic molecule containing CMS candidate genes that had been generated by rearrangements via microhomology-mediated recombination using short sequences overlapped between sequence elements (ranging from 5 to 40 bp; Figure 4) and/or NHEJ of sequence elements from diverse sources might be maintained at low copy number even in normal pepper lines. If the suppression of ectopic recombination is released under certain conditions, amplification of the CMS-specific DNA structure containing *orf507* and ψ*atp6-2* might occur by recombination-dependent replication via intermediate-sized repeat sequences around these region. A pair of intermediate-sized repeats (R21) of which one copy is located downstream of *orf507* and the other downstream of ψ*atp6-2* might be candidates for mediating this procedure. However, prediction of the precise mechanism of the rearrangement is limited by the lack of information on the evolutionary relationship between the CMS cytoplasm and a normal type cytoplasm in Jeju. In fact, the corresponding DNA region found in another type of normal cytoplasm from CM334 showed structural differences when compared to Jeju implying the presence of multiple cytoplasm types that have undergone different levels of rearrangement (Figure 4). Analyses of the mtDNA region containing *orf507*-ψ*atp6-2* from different cytoplasms of pepper may provide clues to the detailed steps of the rearrangements. In addition, further analysis using high-coverage paired-end sequencing may facilitate identification of possible structures of subgenomic molecules to elucidate dynamic processes related to origin of CMS in *Capsicum*.

Considering the specific characteristics of the CMS-associated region in pepper, we performed analysis of the organization of syntenic sequence blocks, repeat distribution, and localization of CMS-associated genes in six additional CMS mitochondrial genomes from other species. In all of the cases, CMS genes were located at the edge of considerably long CMS-specific sequences between syntenic blocks and close to intermediate-sized repeat sequences or on the repeat sequence itself (e.g., CMS-S in maize). None of CMS genes originated by the fusion of sequences that are exist on predominant subgenomic molecules of male-fertile lines nor by small insertions or deletions on pre-existing sequences. These findings fit well with the notion that subgenomic structure containing CMS genes might originate from multiple rearrangements mediated by microhomology-mediated recombination or NHEJ to create novel DNA sequence regions and copy number increases due to recombination via adjacent repeat sequences. Proximity of pre-existing low copy-number CMS genes to intermediate-sized repeat sequences might be the prerequisite to ensure amplification of these sequences required for the induction of CMS as discussed by Davila et al. [7]. The close localization of CMS genes to syntenic sequence blocks might be due to the need for sequence elements required for transcription of a chimeric *orf*. In Arabidopsis, the majority of chimeric *orfs* were shown not to be transcribed [51]. This implies that utilization of promoters in conserved regions might be requisite for the transcription of *orf*s. These common features of genomic environment of CMS-associated genes can be clues to understand the evolution of CMS as well as provide a strategy to screen for unknown CMS-gene candidates by comparative genomics approaches.

## Conclusions

The complete mitochondrial genome sequences of pepper were obtained in CMS and male-fertile lines. A large portion of the intergenic sequences in the pepper lines could not be aligned with the mitochondrial genome of *Nicotiana tabacum*, which is a member of the same family (Solanaceae), whereas sequences and clustering patterns of genes were largely conserved. In the comparison between mitochondrial genomes of CMS and male-fertile pepper lines, however, most genome sequences could be aligned although syntenic sequences were divided into eighteen sequences blocks that were generated by rearrangements in intergenic regions. The CMS candidate genes *orf507* and ψ*atp6-2* were located on the edges of CMS-specific sequence segments that were between syntenic sequence blocks. The presence of many repeat sequences and connection of sequence segments overlapped each other by a few nucleotides implied that extensive rearrangements by homologous recombination and/or NHEJ might be involved in evolution and substoichiometric shift of this region. Extended investigation using CMS-associated genes identified in other species revealed that these genes are specifically localized near edges of CMS-specific DNA regions and intermediate or large-sized repeat sequences indicating the evolution of CMS-associated genes might involve the common mechanism.

## Electronic supplementary material

Additional file 1: **Sequencing and contig assembly results.** (PDF 11 KB)

Additional file 2: **Alignment of mitochondrial genomes of pepper and tobacco.** (PDF 11 KB)

Additional file 3: **Distribution of sequence blocks syntenic between FS4401 and tobacco or Jeju and tobacco (>2 kb; >95%) on FS4401, Jeju, and tobacco mitochondrial genomes.** (PDF 184 KB)

Additional file 4: **Localization of syntenic sequences blocks (>2 kb; > 95%) and size of gap or overlapping sequences between blocks on FS4401 mtDNA.** (PDF 24 KB)

Additional file 5: **Localization of syntenic sequence blocks (>2 kb; > 95%) and size of gap or overlapping sequences between blocks on Jeju mtDNA.** (PDF 24 KB)

Additional file 6: **mtDNA** ***ORF*** **s with polymorphism or unique to one pepper line.** (PDF 14 KB)

Additional file 7: **Repeated sequences around** ***orf507*** **and ψ** ***atp6-2.*** (PDF 26 KB)
