# Supplementary material for: Extensive structural variations between mitochondrial genomes of CMS and normal peppers (Capsicum annuum L.) revealed by complete nucleotide sequencing
Source: BMC Genomics. 2014 Jul 4;15(1):561. doi: 10.1186/1471-2164-15-561 (PMC4108787; doi:10.1186/1471-2164-15-561)
Supplement: Supplementary file 1 — Additional file 1: Sequencing and contig assembly results. (PDF 11 KB) [file 12864_2014_6266_MOESM1_ESM.pdf]

Additional file 1. Sequencing and contig assembly results

Categories	FS4401	Jeju
Total length of sequences analyzed by 454 GS-FLX (bp)	58,113,817	64,752,391
Average length of sequences analyzed by 454 GS-FLX (bp)	247	249
No. mtDNA contigs assembled by Newbler2.0 (>1kb)	33	40
Total length of mtDNA from contigs (>1kb) (bp)	439,940	451,255
No. mtDNA contigs assembled by ABI3700 sequencing (mate-pair) and PCR analysis	12	12
Length of complete mtDNA contig	507,452	511,530
