# Supplementary material for: Extensive structural variations between mitochondrial genomes of CMS and normal peppers (Capsicum annuum L.) revealed by complete nucleotide sequencing
Source: BMC Genomics. 2014 Jul 4;15(1):561. doi: 10.1186/1471-2164-15-561 (PMC4108787; doi:10.1186/1471-2164-15-561)
Supplement: Supplementary file 2 — Additional file 2: Alignment of mitochondrial genomes of pepper and tobacco. (PDF 11 KB) [file 12864_2014_6266_MOESM2_ESM.pdf]

Additional file 2. Alignment of mitochondrial genomes of pepper and tobacco. mtDNA sequences (bp) that could be aligned between two mitochondrial genomes of pepper and one of tobacco by BLASTN algorithm. In parenthesis is the percent coverage of aligned sequences in the mtDNA sequences of the pepper or tobacco line of that column when analyzed against the mtDNA sequence of the line in the corresponding row.

Line	FS4401	Jeju	*Nicotiana tabacum*
FS4401	-	501,474 (98.0%)	237,688 (55.2%)
Jeju	482,589 (95.1%)	-	236,554 (54.9%)
Nicotiana tabacum	234,760 (46.3%)	232,382 (45.4%)	-
