# Supplementary material for: Extensive structural variations between mitochondrial genomes of CMS and normal peppers (Capsicum annuum L.) revealed by complete nucleotide sequencing
Source: BMC Genomics. 2014 Jul 4;15(1):561. doi: 10.1186/1471-2164-15-561 (PMC4108787; doi:10.1186/1471-2164-15-561)
Supplement: Supplementary file 4 — Additional file 4: Localization of syntenic sequences blocks (>2 kb; > 95%) and size of gap or overlapping sequences between blocks on FS4401 mtDNA. (PDF 24 KB) [file 12864_2014_6266_MOESM4_ESM.pdf]

Additional file 4. Localization of syntenic sequences blocks (> 2 kb; > 95%) and size of gap or overlapping sequences between blocks on FS4401 mtDNA

Syntenic block	Length (bp)	Direction	Start site	End site	Gap size [a] (bp; %) [b]	Overlapping sequence size (bp) [c]
block 9	25,275	+	501,287	19,109	4579 (12.64)	-
block 15	78,882	-	23,689	102,570	-	7413
block 2	74,692	-	95,158	169,849	166 (0.46)	-
block 6	4,738	+	170,016	174,753	375 (1.03)	-
block 1'	9,822	+	175,129	184,950	-	6
block 14	11,852	-	184,945	196,796	-	22
block 5	35,959	+	196,775	232,733	-	8
block 16	17,024	+	232,726	249,749	7908 (21.83)	-
block 8	6,016	+	257,658	263,673	17650 (48.71)	-
block 3	20,756	+	281,324	302,079	3323 (9.17)	-
block 11	27,874	+	305,403	333,276	1595 (4.40)	-
block 7	8,478	+	334,872	343,349	-	76
block 10	2,906	+	343,274	346,179	37 (0.10)	-
block 4	28,796	+	346,217	375,012	-	34
block 13	47,000	-	374,979	421,978	-	65
block 12	42,021	+	421,914	463,934	205 (0.57)	-
block 6	4,738	+	464,140	468,877	375 (1.03)	-
block 1	32,015	+	469,253	501,267	19 (0.05)	-

[a] The length of the gap between the indicated block and the one described in the next row.
[b] The ratio of the given gap sequence to the total size of gap sequences is shown as a percentage.
[c] The sequence overlap with the block described in the next row.
