# Supplementary material for: Extensive structural variations between mitochondrial genomes of CMS and normal peppers (Capsicum annuum L.) revealed by complete nucleotide sequencing
Source: BMC Genomics. 2014 Jul 4;15(1):561. doi: 10.1186/1471-2164-15-561 (PMC4108787; doi:10.1186/1471-2164-15-561)
Supplement: Supplementary file 5 — Additional file 5: Localization of syntenic sequence blocks (>2 kb; > 95%) and size of gap or overlapping sequences between blocks on Jeju mtDNA. (PDF 24 KB) [file 12864_2014_6266_MOESM5_ESM.pdf]

Additional file 5. Localization of syntenic sequence blocks (> 2 kb; > 95%) and size of gap or overlapping sequences between blocks on Jeju mtDNA

Syntenic block	Length (bp)	Direction	Start site	End site	Gap size [a] (bp; %) [b]	Overlapping sequence size (bp) [c]
block 1	32029	+	501700	22198	1550 (5.58)	-
block 2	74739	-	23749	98487	45 (0.16)	-
block 3	20781	+	98533	119313	-	51
block 4	28836	+	119263	148098	2 (0.01)	-
block 5	35993	+	148101	184093	10467 (37.71)	-
block 6'	4693	+	194561	199253	-	32
block 7	8483	+	199222	207704	4088 (14.73)	-
block 8	6017	+	211793	217809	-	438
block 9	25281	-	217372	242652	2359 (8.5)	-
block 6	4739	+	245012	249750	-	32
block 7	8483	+	249719	258201	3931 (14.16)	-
block 10	2905	+	262133	265037	-	16
block 11	27994	+	265022	293015	30 (0.11)	-
block 12	42069	+	293046	335114	-	18
block 13	47126	-	335097	382222	3781 (13.62)	-
block 4'	7602	+	386004	393605	-	32
block 14	11823	-	393574	405396	-	1182
block 15	78971	-	404215	483185	-	40
block 16	17052	+	483146	500197	1502 (5.41)	-

[a] The length of the gap between the indicated block and the one described in the next row.
[b] The ratio of the given gap sequence to the total size of gap sequences is shown as a percentage.
[c] The sequence overlap with the block described in the next row.
