# Supplementary material for: Extensive structural variations between mitochondrial genomes of CMS and normal peppers (Capsicum annuum L.) revealed by complete nucleotide sequencing
Source: BMC Genomics. 2014 Jul 4;15(1):561. doi: 10.1186/1471-2164-15-561 (PMC4108787; doi:10.1186/1471-2164-15-561)
Supplement: Supplementary file 6 — Additional file 6: mtDNA ORF s with polymorphism or unique to one pepper line. (PDF 14 KB) [file 12864_2014_6266_MOESM6_ESM.pdf]

Additional file 6. mtDNA *ORF*s with polymorphism or unique to one pepper line. The number of *ORF*s in each category is indicated in parenthesis.

Category	*ORF*s unique to FS4401	*ORF*s unique to Jeju
SNPs	*orf104e, orf112c, orf133d, orf158, orf162, orf204a, orf257, orf293b, orf337, orf353, orf473, orf484, orf675* (13)	*orf112b, orf133, orf158,orf162 orf204a, orf257, orf337, orf353, orf473, orf484, orf675* (11)
In frame Indel	*orf126, orf127, orf132c, orf454* (4)	*orf129, orf130* (2)
Frame shift (+SNPs)	*orf102m, orf107d, orf110d, orf111c, orf115c, orf115d, orf119b, orf131b, orf132d , orf133d, orf149, orf152d, orf165, orf166b, orf190, orf204a* (16)	*orf101a, orf111b, orf112a, orf226 , orf261, orf277 , orf430 , orf498 , orf585* (9)
Rearrangement	*orf100d, orf108a, orf141, orf507, orf244, orf262, orf300, orf338* (8)	*orf102h, orf110c, orf125g,orf470, orf881* (5)
Specific to indicated mitochondrial genome	*orf100c, orf102l, orf119c, orf132e* (4)	*orf103a, orf104b, orf481* (3)
Total number of *ORF*s	45	30

[a] The number of *ORF*s with SNPs were not identical between two lines because *ORF*s that contains SNP on start or stop codons were defined only in one line.
