# Supplementary material for: Extensive structural variations between mitochondrial genomes of CMS and normal peppers (Capsicum annuum L.) revealed by complete nucleotide sequencing
Source: BMC Genomics. 2014 Jul 4;15(1):561. doi: 10.1186/1471-2164-15-561 (PMC4108787; doi:10.1186/1471-2164-15-561)
Supplement: Supplementary file 7 — Additional file 7: Repeated sequences around orf507 and ψ atp6-2. (PDF 26 KB) [file 12864_2014_6266_MOESM7_ESM.pdf]

Additional file 7. Repeated sequences around *orf507* and *ψatp6-2*.

Repeat name[a]	Repeat length	Similarity between repeats in FS4401 (%)	Presence (+) or absence (-) in Jeju[b]
R19	132	98	+
Ra	159	93	+
R21	140	95	+
Rb	81	96	-

[a]The repeat sequence of FS4401 are found in Jeju as single copies
