# Supplementary material for: Extensive structural variations between mitochondrial genomes of CMS and normal peppers (Capsicum annuum L.) revealed by complete nucleotide sequencing
Source: BMC Genomics. 2014 Jul 4;15(1):561. doi: 10.1186/1471-2164-15-561 (PMC4108787; doi:10.1186/1471-2164-15-561)
Supplement: Supplementary file 3 — Additional file 3: Distribution of sequence blocks syntenic between FS4401 and tobacco or Jeju and tobacco (>2 kb; >95%) on FS4401, Jeju, and tobacco mitochondrial genomes. (PDF 184 KB) [file 12864_2014_6266_MOESM3_ESM.pdf]

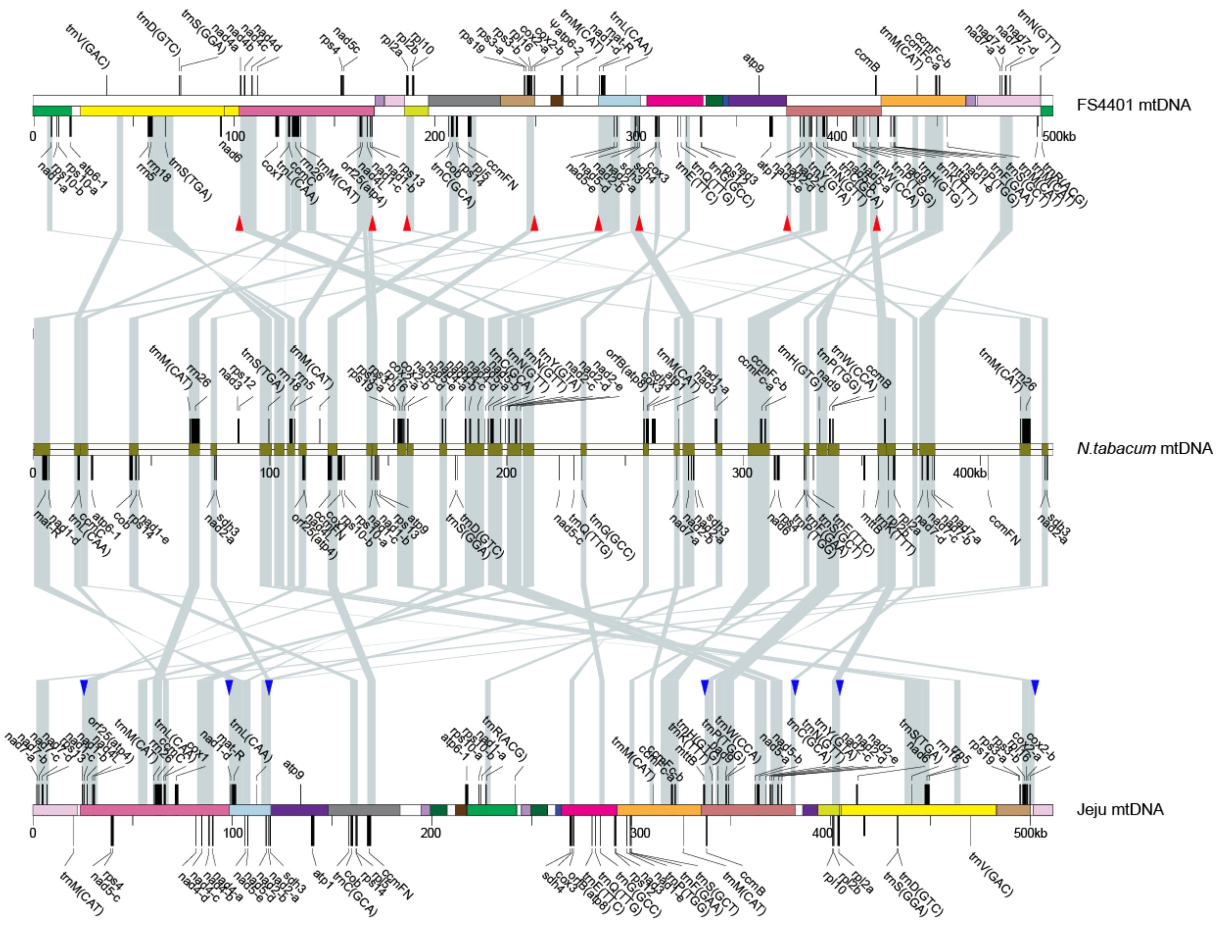

Additional file 3. Distribution of sequence blocks syntenic between FS4401 and tobacco or Jeju and tobacco (>2kb; >95%) on FS4401, Jeju, and tobacco mitochondrial genomes. The DNA blocks in the standard direction was represented on the upper lane and blocks in opposite direction was on shown on the lower lane.
